# Relationship between an editor in chief's commentary publications and the impact factor of cardiovascular medicine journals

**DOI:** 10.5195/jmla.2021.1145

**Published:** 2021-07-01

**Authors:** Alex Salazar, Michael Joseph Berry

**Affiliations:** 1 salaas17@wfu.edu, Department of Health and Exercise Science, Wake Forest University, Winston-Salem, NC; 2 berry@wfu.edu, Professor of Health and Exercise Science, Department of Health and Exercise Science, Wake Forest University, Winston-Salem, NC

**Keywords:** impact factor, Eigenfactor, editorial comment, cardiology

## Abstract

Journal impact factor (IF) inflation is suggested as a problem resulting from commentaries published by the editors in chief (EiCs) of their respective journals. However, it is unclear whether this is a systemic problem across the top thirty cardiovascular medicine journals. Therefore, the purpose of this investigation was to examine the relationship between the number of commentaries written by an EiC and their journal's IF and Eigenfactor (Ef). Utilizing Spearman rank partial correlations controlling for length of service as the EiC, significant moderate correlations were found between the number of commentaries and the number of first-author commentaries by the EiC and the IF of their journal (r=0.568, *p*=0.001 and r=0.504, *p*=0.005; respectively). A weak but still significant correlation was found between the number of commentaries by the EiC and the Ef of their journal (r=0.431, *p*=0.020). The reason for these correlations is unclear, and whether the methodology used to compute the IF and Ef should be modified needs further research.

## INTRODUCTION

A common method for determining the quality of a journal is the Journal Citation Reports' impact factor (IF) [1]. Developed in the 1960s, the IF was used as a tool for selecting journals to include in the Science Citation Index, the predecessor to the Clarivate Web of Science database, and was eventually used as an acquisitions tool by libraries [2, 3]. The IF is the number of times a journal's articles have been cited in other articles in the two previous years divided by the total number of articles published by the journal during the same time period. For example, an IF of ten means that, on average, each article published by a journal in the previous two years had been cited ten times [1].

Due to the simplicity of calculating the IF, it is possible to manipulate the score. Because the IF is simply a mean score, it can be skewed by outliers. Even one highly cited paper can significantly boost a journal's IF [4, 5]. Another manipulation of the IF is through author or journal self-citation or both, which allows authors and journals to bolster the reputation of their works through repeated self-referencing. A study examining the relationship between self-citation rate and journal IF in the field of plastic surgery found that the two were significantly correlated [6]. Self-citation is also problematic within cardiovascular journals [7]. The Eigenfactor (Ef) is another bibliometric indicator that is touted as a solution to problems with the IF. The Ef, which is calculated based on the number of times articles published in the last five years have been cited [8], accounts for self-citation by removing the influence of citations to other articles in the same journal [9, 10].

While there is evidence of journal self-citation in the cardiovascular sciences, it is not clear what role editors in chiefs (EiCs) have in this problem [11]. EiCs often publish commentary pieces, typically drawing attention to or criticizing other academic works. These pieces are typically short, lack an abstract, and serve as a form of communication between the EiC and readers. Commentaries written by EiCs may increase their journal's IF by being cited frequently and citing other articles from the parent journal [12, 13]. The use of commentaries or editorials written by EiCs has been identified as a technique that could manipulate journal IFs. These pieces are not counted in the denominator but are counted in the numerator of the IF calculation, allowing potential manipulation of the IF [14].

The primary aim of this study was to determine the relationship between the number of commentaries written by an EiC and the IF of their journal. A secondary aim was to determine the relationship between the total number of publications by an EiC and the IF of their journal to account for the full influence of an individual editor's contributions to their field on their journal's IF. Tertiary aims were to determine whether the EiC being a first author influenced these correlations and to determine if an EiC's publication record also correlated with their journal's Ef.

## METHODS

Utilizing the Journal Citation Reports database from Clarivate Analytics, we selected the top thirty cardiac and cardiovascular systems journals based on their IF in the year 2019. Cardiology was selected because of previous research indicating problems with self-citation in this field [7, 11]. Table 1 shows the journals included in this analysis, the IF and Ef at the time of this analysis (2020), and the number of commentaries by EiCs published in their journal.

**Table 1:** Cardiology journals' IF, Ef, and number of commentaries by the EiC

Rank	Journal Title	IF	Ef	Commentaries by EiC
1	*Circulation*	23.603	0.20502	59
2	*European Heart Journal*	22.673	0.14062	352
3	*Journal Of The American College Of Cardiology*	20.589	0.19028	61
4	*Nature Reviews Cardiology*	20.260	0.02113	162
5	*Circulation Research*	14.467	0.07147	0
6	*JAMA Cardiology*	12.794	0.03011	67
7	*JACC-Cardiovascular Imaging*	12.740	0.02755	42
8	*Basic Research In Cardiology*	11.981	0.00638	123
9	*European Journal Of Heart Failure*	11.627	0.02870	83
10	*JACC-Heart Failure*	8.750	0.01918	58
11	*JACC-Cardiovascular Interventions*	8.432	0.03733	57
12	*Cardiovascular Research*	8.168	0.01995	1
13	*Journal Of Heart And Lung Transplantation*	7.865	0.02814	81
14	*Cardiovascular Diabetology*	7.332	0.01139	16
15	*Progress In Cardiovascular Diseases*	6.763	0.00834	118
16	*European Heart Journal-Cardiovascular Pharmacotherapy*	6.696	0.00164	28
17	*Circulation-Heart Failure*	6.033	0.01849	21
18	*European Journal Of Preventive Cardiology*	5.864	0.01537	56
19	*Heart Rhythm*	5.731	0.02862	45
20	*Circulation-Cardiovascular Imaging*	5.691	0.01632	15
21	*Journal Of The American Society Of Echocardiography*	5.508	0.01823	20
22	*Circulation-Cardiovascular Interventions*	5.493	0.01814	23
23	*Journal Of Cardiovascular Magnetic Resonance*	5.361	0.01112	5
24	*Clinical Research In Cardiology*	5.268	0.00728	21
25	*Heart*	5.213	0.03014	200
26	*Circulation-Cardiovascular Quality And Outcomes*	5.071	0.01435	40
27	*Canadian Journal Of Cardiology*	5.000	0.01763	20
28	*European Heart Journal-Cardiovascular Imaging*	4.841	0.02311	7
29	*Trends In Cardiovascular Medicine*	4.755	0.00392	0
30	*Revista Espanola De Cardiologia*	4.642	0.00461	14

JAMA – *Journal of the American Medical Association*; JACC – *Journal of the American College of Cardiology*.

The EiCs of journals were found using the journals' websites. PubMed was used to determine the following values for each EiC: number of commentaries published, number of commentaries for which the EiC was the first author, total number of publications, and total number of publications for which the EiC was the first author. The number of commentaries published was restricted to the EiC's journal, whereas total number of publications included all items published regardless of the journal. Commentaries were defined as any article written by the EiC published in their own journal that specifically said “no abstract” in PubMed. The approximate date of the appointment of each EiC was also determined to control for variation in publication counts based on the length of time each EiC served. If a journal had co-EiCs, the first EiC that appeared alphabetically was used.

Data were analyzed using SPSS version 26. Data were tested for normality using the Shapiro-Wilk test. Partial correlations were computed using Spearman rank correlations. EiCs' publications were correlated with their journals' IF and Ef while controlling for the number of years the EiC had served to statistically remove the influence of length of EiC experience. Comparisons between correlation coefficients were performed by converting each correlation coefficient into a z-score using Fisher's r-to-z transformation. Asymptotic covariance of the estimates were computed, and these quantities were used in an asymptotic z-test [15]. Significance was set at *p*<0.05.

## RESULTS

For the journals included in this analysis, IFs ranged from 4.642 to 23.603, with a median IF of 6.730 and an interquartile range of 5.338 to 12.171. Efs ranged from 0.00164 to 0.20502, with a median Ef of 0.01884 and an interquartile range of 0.01132 to 0.02905. Spearman rank correlations between journal IF and Ef and their EiC's publication records are shown in Table 2.

**Table 2:** Spearman correlations between journal IF and Ef and their EiC's publication records

	Publications	First-author publications	Commentaries	First-author commentaries
IF	Correlation	0.197	0.377	0.568	0.504
	p value	0.305	0.044	0.001	0.005
Ef	Correlation	0.283	0.366	0.431	0.301
	p value	0.137	0.051	0.020	0.112

Note: All correlations are Spearman rank partial correlations controlling for years served as EiC.

Considering the IF, no significant correlation was found between the IF of a journal and the total number of publications by its EiC. However, weak to moderate significant correlations were found between the IF and the numbers of first-author publications by the EiC, commentaries by the EiC, and first-author commentaries by the EiC. Considering the Ef, the only significant correlation was between the Ef of a journal and the number of commentaries by its EiC. The magnitudes of the IF and Ef correlations with the number of commentaries and first-author commentaries by the EiC were not significantly different (*p*=0.261 and 0.193, respectively).

## DISCUSSION

In this study, we analyzed correlations between various categories of EiCs' publications and scientometric indices (i.e., IF and Ef) of their journal while controlling for the number of years served by the EiC. We found weak to moderate, statistically significant correlations between the number of commentaries published by EiCs and these scientometric indices.

Previous literature shows an association between journal self-citation and the IF [6]. Journal self-citation typically focuses on a single paper that can boost an IF when published. Opthof explains that something as mundane as the *Journal of the American College of Cardiology* releasing a “Highlights of the Year” paper would allow their IF to rise by 0.309. This rise in IF would allow them to overtake *Circulation*, which at the time had the highest IF among cardiovascular journals, establishing the *Journal of the American College of Cardiology* as the journal with the highest IF in 2010 [7]. While journal self-citation has been identified as a technique that can be used to intentionally inflate the IF of journals, the fact that we found positive correlations between the EiCs' commentaries and IF and Ef does not provide evidence that EiCs are using self-citation or journal self-citation to manipulate the IF or Ef of their journals. Rather, these correlations may simply be a result of the fact that EiCs of journals with higher IFs publish more commentaries in an attempt to provide greater insight into the articles published in their journals.

Heneberg performed a citation analysis of publications appearing in eleven arbitrarily selected prominent general science and biomedical journals [16], which showed that original data-based research accounted for 12% to 79% of items published by the journals, whereas 11% to 44% of items published were considered editorial materials. Heneberg reports that these editorial materials, along with commissioned opinion articles in the parent journal and those published in other journals, accounted for up to 30% of the total citations in these top tier journals, thus affecting their IF and Ef. Furthermore, over a three-year period from 2009 to 2011, these journals reported a similar percentage of citations for data-based articles and reviews versus other editorial materials. Heneberg recommends excluding journal self-citations and certain document types (including editorial comments) from the calculation of the IF and Ef in an effort to limit both intentional and unintentional manipulation of these scientometric indices.

Because of the problems of journal self-citation and the use of different types of published items to calculate the numerator and denominator of the IF, Liu et al. suggest a number of solutions [14]. These solutions include devising new scientometric indices, such as the SCImago Journal Rank and Science Normalized Impact Per Paper indicators based on the Scopus database; computing the IF based on the type of article or item published by a given journal; and redefining the types of articles or items to be included in the numerator and denominator of the IF [14]. They evaluated the effect of adjusting the numerator and denominator of the IF based on the type of item published in ophthalmologic or mathematical journals. Their results showed that changes in the way in which the IF is calculated had an effect for ophthalmologic journals and thus may be valuable; however, the change did not seem to be useful when evaluating mathematic journals. Whether changes in the computation of the IF for cardiovascular medicine journals would be valuable is unclear.

Some limitations of the current study should be noted. Correlations were used to examine the relationship between EiCs' publications and their journals' IF and Ef. As such, a causal relationship between the two cannot be established. A few of the journals had two EiCs, and only the first one alphabetically listed was used for data collection. Due to the large number of publications analyzed, individual confirmation of whether each article was in fact a commentary was not performed—rather, whether the article had “no abstract” listed in PubMed was the criteria for denoting a commentary piece. Thus, a more precise and efficient method of enumerating commentaries is needed, such as developing a coding script to automate data collection. We examined the top cardiovascular medicine journals, but our analysis was not sensitive enough to determine whether particular journals engaged in excessive self-citation. A more detailed analysis of each individual journal would be required to obtain this information.

In conclusion, we found significant moderate correlations between a journal's IF and the number of all commentaries and first-author commentaries published by its EiC. Also, a weak but statistically significant correlation was found between a journal's Ef and the number of commentaries published by its EiC. Whether the methodology used to compute the IF and Ef for cardiovascular medicine journals should be modified requires further research.

## Data Availability

Data associated with this article are available in the Figshare repository at: https://figshare.com/articles/dataset/JMLA_1145_Data_ sav/13725835.
